# Transcriptome Analyses in Adult Olive Trees Indicate Acetaldehyde Release and Cyanide-Mediated Respiration Traits as Critical for Tolerance against *Xylella fastidiosa* and Suggest AOX Gene Family as Marker for Multiple-Resilience

**DOI:** 10.3390/pathogens13030227

**Published:** 2024-03-05

**Authors:** Birgit Arnholdt-Schmitt, Debabrata Sircar, Shahid Aziz, Thais Andrade Germano, Karine Leitão Lima Thiers, Carlos Noceda, Revuru Bharadwaj, Gunasekaran Mohanapriya, José Hélio Costa

**Affiliations:** 1 Non-Institutional Competence Focus (NICFocus) ‘Functional Cell Reprogramming and Organism Plasticity’ (FunCROP), Coordinated from Foros de Vale de Figueira, 7050-704 Alentejo, Portugal; debabrata.sircar@bt.iitr.ac.in (D.S.); shahidaziz.cl@gmail.com (S.A.); tandradeg@gmail.com (T.A.G.); kkthiers@gmail.com (K.L.L.T.); cana293@yahoo.es (C.N.); bharat.revuru@gmail.com (R.B.); mohanapriya.biotechnology@gmail.com (G.M.); 2 Functional Genomics and Bioinformatics Group, Department of Biochemistry and Molecular Biology, Federal University of Ceará, Fortaleza 60451-970, Brazil; 3 Biosciences and Bioengineering Department, Indian Institute of Technology Roorkee, Roorkee 247667, India; 4 Cell and Molecular Biotechnology of Plants (BIOCEMP)/Industrial Biotechnology and Bioproducts, Departamento de Ciencias de la Vida y de la Agricultura, Universidad de las Fuerzas Armadas-ESPE, Sangolquí 171103, Ecuador; 5 Facultad de Ingeniería, Universidad Estatal de Milagro (UNEMI), Guayas 091050, Ecuador

**Keywords:** stress alleviation, alternative respiration, secondary metabolism, fungal endophytes, remote sensing, deep trait markers, functional marker development

## Abstract

*Xylella fastidiosa* (*Xf*) is a global bacterial threat for a diversity of plants, including olive trees. However, current understanding of host responses upon *Xf*-infection is limited to allow early disease prediction, diagnosis, and sustainable strategies for breeding on plant tolerance. Recently, we identified a ***ma***jor ***c***omplex ***t***rait for ***e***arly ***d***e novo programming, named *CoV-MAC-TED*, by comparing early transcriptome data during plant cell survival with SARS-CoV-2-infected human cells. This trait linked ROS/RNS balancing during first hours of stress perception with increased aerobic fermentation connected to alpha-tubulin-based cell restructuration and control of cell cycle progression. Furthermore, our group had advanced concepts and strategies for breeding on plant holobionts. Here, we studied tolerance against *Xf*-infection by applying a *CoV-MAC-TED*-related gene set to (1) progress proof-of-principles, (2) highlight the importance of individual host responses for knowledge gain, (3) benefit sustainable production of *Xf*-threatened olive, (4) stimulate new thinking on principle roles of secondary metabolite synthesis and microbiota for system equilibration and, (5) advance functional marker development for resilience prediction including tolerance to *Xf*-infections. We performed hypothesis-driven complex analyses in an *open access* transcriptome of primary target xylem tissues of naturally *Xf*-infected olive trees of the *Xf*-tolerant cv. Leccino and the *Xf*-susceptible cv. Ogliarola. The results indicated that cyanide-mediated equilibration of oxygen-dependent respiration and carbon-stress alleviation by the help of increased glycolysis-driven aerobic fermentation paths and phenolic metabolism associate to tolerance against *Xf*. Furthermore, enhanced alternative oxidase (AOX) transcript levels through transcription *Gleichschaltung* linked to quinic acid synthesis appeared as promising trait for functional marker development. Moreover, the results support the idea that fungal endophytes strengthen *Xf*-susceptible genotypes, which lack efficient AOX functionality. Overall, this proof-of-principles approach supports the idea that efficient regulation of the multi-functional AOX gene family can assist selection on multiple-resilience, which integrates *Xf*-tolerance, and stimulates future validation across diverse systems.

## 1. Introduction

*Xylella fastidiosa* (*Xf*) is a Gammaproteobacteria with a wide plant host range. In many hosts, *Xf* behaves as endophyte without inducing harmful symptoms. However, in other plant species, such as *Olea europaea*, *Vitis vinifera* and several important citrus plants, infection with *Xf* can become virulent with disastrous pathogenic effects [1]. Causality of these differences among host responses is not well understood. Nevertheless, breeding efforts on higher host tolerance assisted by neutral marker sequences are promising [2,3]. however, the search for more efficient functional marker candidates for sustainable *Xf*-tolerance or -resistance that might be used across populations and species and, which could improve also general resilience of existing cultivars, is still in its infancy [1,4]. climate conditions [5] and the host’s internal and external microbiota [6,7], are studied as potential influencers of *Xf*-threatened plant performance.

*Xf* is introduced directly into the xylem tissue by hemipteran insect vectors. thus, the host’s immune response in primarily targeted vascular cells is the first barrier against bacterial colonization [6]. consequently, early host cell response in *Xf*-infected xylem cells can be compared to virus-infected human nasal epithelial cells and abiotic stress-affected plant cells to validate principles encountered for ‘stress-induced cell reprogramming’. 

Recently, we identified *CoV-MAC-TED* as a ***ma***jor ***c***omplex ***t***rait for ***e***arly ***d***e novo cell programming. This trait was found promising to identify similar and differential early responses upon SARS-CoV-2 variants and influenza H3N2 in human primary target nasal cells of diverse individual origins [8,9]. *CoV-MAC-TED* was identified by help of an interdisciplinary approach that integrated studies on successful plant cell survival and SARS-CoV-2-infected cell systems [10]. Results of these studies pointed to the importance of adaptive ROS/RNS balancing during first hours of stress perception and its connection to increased aerobic fermentation, which was further linked to alpha-tubulin-based cell restructuration and control of cell cycle progression [11,12]. Subsequently, we observed stress-induced *Gleichschaltung* of accumulated transcripts from ROS-balancing marker genes, such as ASMTL in human cells and AOX in plant cells, which suggested the time-dependent control of transcript diversity and associated sequence polymorphisms in these genes could be developed as trait *per se* for predicting healthy resilience [13,14,15].

Infection with *Xf* results in local and systemic effects related to chronic oxidative stress and disease development [16]. The main macroscopic symptom, which finally can become lethal, is drying of the leaves. Blockage of water transport in xylem seems to be crucial for negative symptoms and lethality [17,18]. However, it is by far not clear, to which degree accumulation of tylose, embolism and bacterial mechanisms, which balance bacterial biofilms versus planktonic solitary cells or small colonies, are the most critical for the disastrous impact on the whole plant organism. In fact, there is no report on a straight forward correlation between symptom severity and bacterial titers [19,20,21]. From all reported observations, we suggest that, once the host response upon *Xf*-infection was induced in primary target cells, all these observed events act in a variable and dynamic way together dependent on the individual plant’s unique overall constitution, and the symptom of dried leaves might not be the result of predictable mono- or poly-factorial key events. 

Vascular tissue as a whole is among the most flexible systems to secure survival of land plants [22,23,24,25]. Individual plants can show high variability for the number, size and distribution of vascular bundles [26]. Furthermore, wound- and pathogen-induced de novo xylem formation was reported [27]. Thus, deep-learning phenotyping of vascular bundles in individual plants could certainly benefit research on xylem-targeting *Xf*-infections [26]. To our knowledge, the role of cambial vascular tissues and the possibility of de novo xylem formation were not, or not sufficiently, explored in host responses to *Xf*-infection. This is in contrast to the important functional role of stem cells, such as xylem cambium, for adaptive robustness of organisms through cell renewal and de novo structuring [23,28]. Also, cambial vascular cells are important for stress perception and its transformation into energy-dependent cell fate decisions through cell cycle activity regulation [29,30].

There is agreement that stress-induced increase in soluble sugars in xylem tissues and ‘feeding’ of glycolysis, in general, play crucial roles for *Xf*-tolerance [17,18,31,32]. Also, glycolysis-dependent phenolic compounds were studied for their appropriateness to indicate *Xf*-resistant performance [33,34,35,36]. In olive, differential synthesis of quinic acid and quercin-3-O-rhamnoside were among the most promising indicators for plant genotype-dependent *Xf*-infection. However, no resistant-related profiles had been encountered. Consequently, it was concluded that presence or absence of phenolic compounds cannot be the only factor for distinguishing tolerant or resistant genotypes, but could improve case-by-case understanding of the mechanisms underlying tolerance (rather than resistance) against *Xf* [36]. In grapevine, phenolic compounds were found to increase initially, but to decline at later stages of disease development, which was suggested to point to limited resources for supporting secondary metabolite production [33].

AOX genes belong to a small key gene family of the alternative respiration pathway, which is encoded in the nucleus, but functions in mitochondria. AOX expression correspondent to its capacity was identified in root and hypocotyl meristems with a link to xylem differentiation [37]. Moreover, NaCl-stress affected AOX expression and at the same time primary xylem differentiation, but peroxidase activity in primary and secondary xylem remained stable [38]. Cellular reprogramming is an energy intensive process. However, Clifton et al. [39] showed that alternative respiration was more variable upon stress than respiration through the COX pathway. AOX acts down-stream and in interaction with oxidative stress signaling- and glycolysis-dependent metabolic changes. The multifunctional role of AOX as switch between sugar-dependent respiration and aerobic fermentation associated to stress-induced cellular reprogramming and related morpho-physiological events is currently better understood and was recently extensively reviewed in Arnholdt-Schmitt et al. [10], Costa et al. [8,11] and Bharadwaj et al. [40] and reviewing shall not be repeated here.

**Figure 1:**
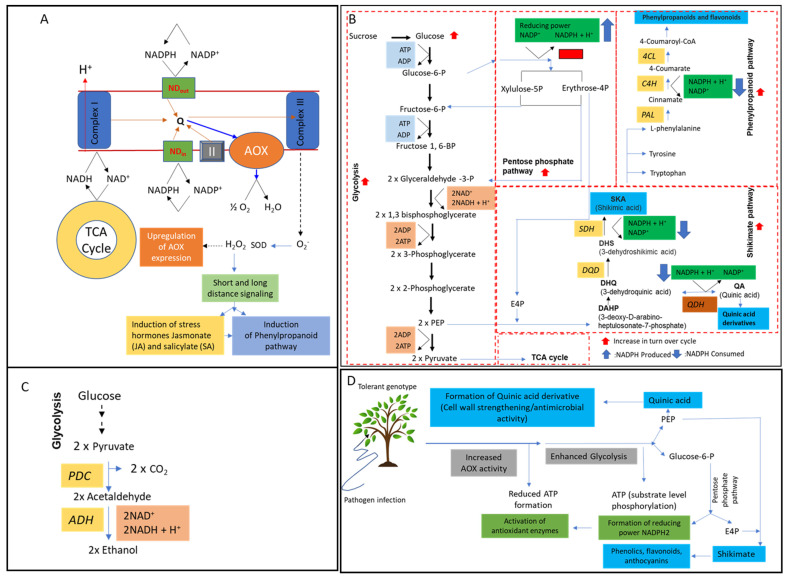
AOX’s role in biotic stress tolerance through metabolic reprogramming. (**A**) By bypassing proton pumps III and IV, electrons from the respiratory electron transport chain (ETC) pass through AOX, creating hydrogen peroxide (H_2_O_2_), a signaling molecule. Phytohormone (JA, SA) biosynthesis and phenylpropanoid metabolism are likely to be upregulated by H_2_O_2_. (**B**) Bypassing proton pumps III and IV through increased AOX activity, ETC induces glycolysis turnover to generate ATP by substrate-level phosphorylation. The glycolytic intermediate glucose-6-phosphate is converted to reducing power NAD(P)H_2_ by entering the pentose phosphate pathway (PPP). To produce DHAP, erythrose-4-phosphate, an intermediate from PPP, is combined with PEP. The conversion of DHAP to shikimic acid (SKA) occurs through the consumption of NAD(P)H_2_. The SKA pathway is used to produce quinic acid derivatives and aromatic amino acids, including phenylalanine, which is converted into phenolics, and flavonoids. (**C**) Increasing the turnover number of glycolysis and preventing pyruvate from entering the TCA cycle by increasing the alcoholic fermentation of pyruvate. (**D**) Summary of how biotic stress induces metabolic reprogramming in plant tissues to produce quinic acid derivatives, phenylpropanoids, antioxidant enzymes, and reducing power. AOX: alternative oxidase; JA: jasmonic acid; SA: salicylic acid; H_2_O_2_: hydrogen peroxide; TCA: tricarboxylic acid cycle; PEP: phosphoenol pyruvate; E4P: erythrose-4-phosphate; PAL: phenylalanine ammonia-lyase; C4H: cinnamate-4-hydroxylase; 4CL: 4-coumaroyl-CoA ligase; DQD: DHQ dehydrogenase; SDH: shikimate dehydrogenase; QDH: quinate dehydrogenase; PDC: pyruvate decarboxylase; ADH: alcohol dehydrogenase: shikimic acid.

In this study, we assumed that the AOX/COX ratio is critical for *Xf*-tolerant plant performance in primary target cells/tissues (see also [41,42,43,44,45]) and hypothesized that this relation can be studied at transcriptome level. We considered our former finding that AOX-inhibition can affect accumulation of soluble and wall-bound phenolics, flavonoids and lignin during de novo differentiation in olive and other plant systems [40,46,47] and suggested also that AOX transcript level regulation will indirectly interfere with phenolic and shikimate metabolite biosynthesis as part of the response to the bacterial threat. Figure 1 points to the rationale of this hypothesis. Additionally, we initiated validating our currently advanced view on the potential role of plant-microbiome systems for stress-relief from high carbon skeletons in tolerant and susceptible genotypes [40].

## 2. Materials and Methods

### 2.1. CoV-MAC-TED-Related Gene Set to Study Early Cellular Reprogramming in Olea Europaea

Genes selected (*CoV-MAC-TED*-related genes) to evaluate early cellular reprogramming in olive under *Xylella fastidiosa* infection (*Xf* infection) corresponded to the enzymes from different cellular metabolic pathways such as: glycolysis [Hexokinase (HXK), Phosphofructokinase (PFK), Enolase (Eno) and Pyruvate kinase (PK)]; ethanol Fermentation [Pyruvate Decarboxylase (PDC) and Alcohol Dehydrogenase (ADH)]; lactic Fermentation [Lactate Dehydrogenase (LDH)]; aerobic respiration/ATP synthesis [Cytochrome c oxidase (COX)]; cyanide detoxification [β-Cyanoalanine synthase (β-CAS)]; alternative respiration pathway [alternative oxidase (AOX)]; energy status-signaling [*Sucrose non*-*fermenting protein kinase* (SNF)]; cell cycle regulation [Target Of Rapamycin (TOR), Transcription factor E2F (E2F)]; oxidative stress signaling [Superoxide Dismutase (SOD), mitochondria-located MnSOD1, NADPH oxidases (RBOH) and Alcohol dehydrogenase2 / S-nitrosoglutathione reductase (ADH2_GSNOR)]; NO production [Nitrate reductase (NR)]; ethanol/acetaldehyde tolerance [D-aminoacyl-tRNA deacylase (GEK1)]; acetaldehyde detoxification [mitochondrial aldehyde dehydrogenase (mtALDH)]; phenolic marker candidates for Xf-tolerance [Quitinate dehydrogenase (QDH), Quercetin-3-O-rhamnoside (3o-GT)]; structural cell organization [alpha-tubulin (alpha-Tub), beta-tubulin (beta-Tub) and gamma-tubulin (gamma-Tub)]; Gluconeogenesis [pyrophosphate-fructose 6-phosphate 1-phosphotransferase (PFP) and phosphoenolpyruvate carboxykinase (PEPCK)]; Gluconeogenesis: *PEP-regeneration phase of the C4 photosynthetic pathway* [pyruvate orthophosphate dikinase (PPDK)]. The accession numbers of all gene members identified in GenBank (NCBI) are shown in Supplementary Table S1.

### 2.2. In Silico Gene Expression Analyses from Olive RNA-seq Data

To advance on the function of early cellular reprogramming in olive trees under *Xf* infection, the gene expression was evaluated using a specific transcriptomic raw data (RNA-Seq) deposited in the Sequence Read Archive (SRA) database from GenBank (NCBI)]. These raw data under BioProject number PRJNA316374 were publicly available by Giampetruzzi et al. [48] from Consiglio Nationale delle Ricerche, Istituto per la Protezione Sostenibile, coordinator Pasquale Saldarelli. Details of this study constituted by 10 olive trees (2 health and 3 *Xf*-infected) from cv. Leccino (*Xf*-tolerant) and cv. Ogliarola (*Xf*-susceptible) are shown in Supplementary Table S2. 

The RNA-seq expression analyses were carried out by mapping the cDNA sequence of each gene against different SRA data experiments through Magic-Blast software [49]. The HTseq program was employed to calculate the number of mapped reads to each gene [50]. Subsequently, the data normalization was performed using the Reads Per Kilobase of transcript per Million of mapped reads (RPKM) method [51] according to the following equation: RPKM = (number of mapped reads × 10^9^)/(number of sequences in each database × number of nucleotides of each cDNA). For enzymes with several gene members, in general, we used the RPKM sum of all genes to estimate the “TOTAL” amount of transcript associated with that enzyme. Data were presented considering olive trees (health and *Xf*-infected) individually or taking into account the means ± standard errors (SE) from health or *Xf*-infected trees of cv. Leccino (*Xf*-tolerant) and cv. Ogliarola salentina (*Xf*-susceptible).

### 2.3. Endophytes Identification in Olive RNA-seq Data

The search of fungal endophytes in olive transcriptomic data was performed using three identification markers: internal transcribed spacer 2 (ITS2), RNA polymerase 1 (RBP1) and beta tubulin (Beta-Tub). For ITS2, we used a set of 256448 ITS2 sequences retrieved from the ITS2 database (https://its2.bioapps.biozentrum.uni-wuerzburg.de, accessed on 25 January 2024) [52]. However, for RBP1 and Beta-Tub, 2380 and 939 fungal sequences were obtained from Genbank (NCBI), respectively. For bacteria endophytes, only 16S rRNA was used as identification markers. Sequences of 25,984 bacterial rRNAs were retrieved from Genbank and used in this analysis. All identification markers were aligned with olive transcriptomic data and the reads obtained were normalized as described in the Section 2.2.

### 2.4. Data Treatment

This study aimed to explore the relationship of accumulated transcripts from a selected gene set to compare individual olive tree performance at a common sampling time point. Therefore, we chose analyzing RNAseq data using RPKM as normalization method (see in Section 2.2). This normalization permits a one-sampling-strategy with subsequent data mining that excludes, or at least minimizes, technical errors since RPKM transcript levels associate the total of reads with the length of each gene during the read mapping step [51]. 

We want to highlight that we interpret our data as ‘real’ observations under the employed conditions involving only small samples, which certainly provide insights that cannot get relevance or not relevance by using significance calculation [8,9,11]. Nevertheless, we calculated also mean values and standard errors (Table S3) to highlight evident differences for infection and cultivars and to enable calculating interaction between both factors. We applied significance calculations at usual *p*-values for biological research as an additional information and aid to appropriately allow wider insights without ignoring the relevance of individuality. Readers are encouraged to making themselves familiar with the current paradigm change related to the usage of statistical significance [53,54,55,56,57].

Statistics were carried out using bifactorial ANOVA to estimate main effects, previous verification of pre-requisites to use parametric analyses. In some cases, Tuckey post-hoc tests were made to estimate interactions. Furthermore, regression analyses were performed, after checking pre-requisites, to explore the strength of general correlation between mainly transcript levels of AOX and the accumulated transcripts of all other genes across all trees (from both cultivars, infected and healthy). InfoStat package and Excel complements were used for statistics.

## 3. Results and Discussion

In the current section, we elaborated the results by following a proof-of-principle approach to validate general utility of the major complex trait *CoV-MAC-TED* for identifying deep traits and candidate genes for functional marker development. Thus, we validated step-by-step whether the obtained results based on transcriptome analyses indicated that *Xf*-infection induced ROS/RNS rebalancing connected to enhanced, glycolysis-related aerobic fermentation, adaptive respiration traits and tubulin-based cell restructuration, which could be linked to the control of cell cycle activity. At the same time, we investigated, whether the targeted gene set could help to discriminate olive trees from two cultivars with differential *Xf*-susceptibility. 

**Figure 2:**
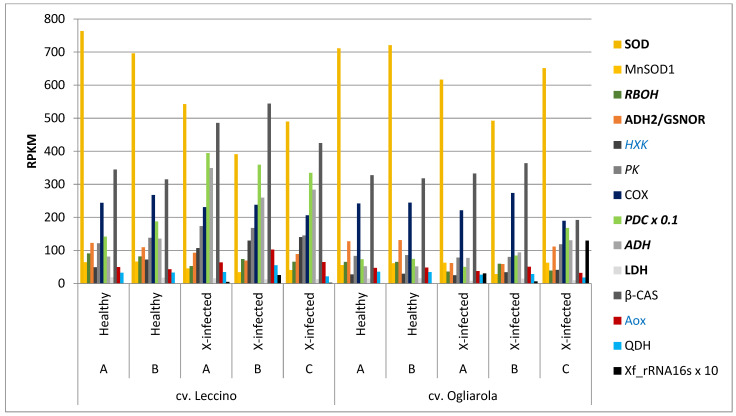
Transcript accumulation of an extended set of CoV-MAC-TED-related genes is shown from xylem tissues of mature shoots of two healthy (A/B) and three *Xylella fastidiosa (Xf)*-infected olive trees (A/B/C) from cv. Leccino (*Xf*-tolerant) and cv. Ogliarola (*Xf*-susceptible). Selected genes represent oxidative stress and ROS/RNS-balancing (SOD, MnSOD1, RBOH, ADH2/GSNOR), glycolysis (HXK, PK), COX-Pathway (COX), alcoholic and lactic fermentation (PDC, ADH, LDH), cyanide detoxification (β-CAS), alternative respiration (AOX) and synthesis of quinic acid (QDH). RPKM: Reads Per Kilobase per Million mapped reads, HXK: Hexokinase, PK: Pyruvate Kinase, COX: cytochrome c oxidase, PDC: Pyruvate decarboxylase, ADH: alcohol dehydrogenase, LDH: Lactate Dehydrogenase, β-CAS: β-Cyanoalanine synthase, AOX: Alternative oxidase, QDH: Quinate dehydrogenase SOD: Superoxide dismutase, RBOH (respiratory burst oxidase homologue): NADPH oxidase, ADH2/GSNOR: S-nitrosoglutathione reductase associated with alcohol dehydrogenase 2 family, Xf_rRNA 16S (×10); 16S ribosomal RNA (10 times) to monitor the bacterial infection. This study was performed in public transcriptome raw data provided by Giampetruzzi et al. [48]. Evident differences between RPKM means, taking into account standard errors for each plant gene (Table S3), are highlighted for main effects of infection (bold) and cultivar (italics). Evident interactions between both factors (infection, cultivar) are highlighted by blue letters and explained in the text.

Figure 2 shows complex transcript level profiles from xylem tissues of mature shoots from two healthy and three *Xf*–infected olive trees of the *Xf*-tolerant cv. Leccino and the *Xf*-susceptible cv. Ogliarola (RPKM values for individual trees and absolute mean RPKM values plus standard errors (SE) are presented in Table S3). The selected gene set relates mainly to *CoV-MAC-TED* (Supplementary Table S1). RNA-Seq data were derived from BioProject PRJNA316374 (Supplementary Table S2).

All trees from both cultivars, which were from *Xf*-affected regions, showed low transcript-levels of *Xf*_rRNA16S. This can be expected to indicate viable states of *Xf* [58], although it cannot judge alone the culturable or non-culturable state of the bacteria [59]. In any case, we observed differences in the degree of infection for individual trees of both cultivars. Tree C of cv. Ogliarola showed highest *Xf*-infection among all trees from both cultivars (12.974 RPKM). The highest *Xf*-infection among the three affected trees from cv. Leccino was observed for tree B (2.535 RPKM). Also, mean *Xf*_rRNA16S transcript accumulation confirmed higher *Xf*-infection for the *Xf*-susceptible cv. Ogliarola cultivar as reported by the group that provided open access to the olive transcriptomic data (Giampetruzzi et al. [48].

### 3.1. Transcript Levels of RBOH, NR, and ADH2/GSNOR Indicated High Oxidative Stress in Healthy Adult Olive Trees and ROS/RNS Rebalancing under Xf-Infection. However, Higher Transcript Levels of SOD in cv. Ogliarola Signaled Higher Oxidative Stress in the Susceptible Cultivar in Xf-Infected Trees

Transcript levels for SOD and RBOH indicated higher ROS-related oxidative stress levels in healthy trees compared to *Xf*-infected trees for both cultivars [healthy trees: SOD 730.01 RPKM for cv. Leccino and 716.05 RPKM for cv. Ogliarola; RBOH 86.22 RPKM for cv. Leccino and 65.01 RPKM for cv. Ogliarola; Xf-infected trees: SOD 474.42 RPKM for cv. Leccino and 586.64 RPKM for cv. Ogliarola; RBOH 64.07 RPKM for cv. Leccino and 44.90 RPKM for cv. Ogliarola Table S3]. Nitrite oxide (NO)—producing nitrate reductase (NR) displayed low transcript levels (thus not shown in Figure 2), but signaled higher transcript accumulation for *Xf*-infected trees vs. healthy trees in both cultivars [healthy trees: 5.53 RPKM for cv. Leccino and 3.27 RPKM for cv. Ogliarola; *Xf*-infected trees: 8.2 RPKM for cv. Leccino and 6.27 RPKM for cv. Ogliarola (Table S3)]. Accordingly, transcript levels of ADH2/GSNOR, which is involved in NO homeostasis, were higher in healthy than in *Xf*-infected trees from both cultivars [healthy trees: 116.07/129.41 RPKM; *Xf*-infected trees: 83.66/77.17 RPKM]. Thus, rebalancing of ROS/RNS was a clearly indicated common response upon *Xf*-infection across both cultivars. Nevertheless, the higher transcript level for SOD in the *Xf*-affected trees in the susceptible cultivar signaled higher overall oxidative stress.

However, we observed individual oxidative stress response patterns upon *Xf*-infection in both cultivars. For example, among the three trees of cv. Leccino, tree B indicated lowest transcript levels for SOD [391.29 RPKM, cultivar mean: 474.42 RPKM], but highest transcript accumulation for RBOH [73.87 RPKM, mean 64.07 RPKM] and NR [12.85 RPKM, mean: 8.2 RPKM] combined with lowest accumulation of ADH2/GSNOR [69.4 RPKM, mean: 83.66 RPKM]. On the other hand, tree C of cv. Ogliarola displayed highest transcript level among all infected trees for ADH2/GSNOR (111.44 RPKM) and lowest NR transcript accumulation (3.53 RPKM) at highest SOD transcript level (651.57 RPKM) among all infected trees from both cultivars and medium or low transcript level for RBOH (39.06 RPKM, mean value for cv. Ogliarola 44.9 RPKM and for cv. Leccino 64.07 RPKM). These data sets indicate concrete evidence of uniqueness in the response of individual trees.

### 3.2. Healthy and Xf-Infected Trees of cv. Leccino Were Distinguished from cv. Ogliarola by Transcript Levels of Regulatory Glycolysis-Related Enzymes and Enzymes Involved in Ethanol Fermentation

Regulatory enzymes HXK and PK, representing first and last step in glycolysis, demonstrated higher transcript levels in cv. Leccino (tolerant) in both healthy trees (means HXK: 60.79 RPKM; PK: 129.93 RPKM; Table S3) compared to those observed in the two healthy trees of cv. Ogliarola (susceptible) (means HXK: 28.41 RPKM, PK: 84.63 RPKM; Table S3). Moreover, in the *Xf*-tolerant cultivar, HXK and PK transcript levels indicated increased glycolysis associated to *Xf*-infection (HXK: 125.58 RPKM; PK: 162.28 RPKM; Table S3). However, in cv. Ogliarola, transcript accumulations for HXK and PK upon *Xf*-infection remained at similar levels in trees A (HXK: 24.66, PK: 78.16 RPKM, Table S3) and B (HXK: 34.07, PK: 80.1 RPKM) compared to both healthy trees (HXK: 27.3/29.53 RPKM, PK: 83.17/86.09 RPKM). Nevertheless, in tree C, which showed highest *Xf*-infection, higher transcript accumulation of HXK (40.87 RPKM) and PK (118.47 RPKM) was observed also in cv. Ogliarola. 

In both cultivars, we found only low transcript levels for glycolysis-related regulatory gene phosphofructokinase (PFK) (Supplementary Figure S1). Despite these low levels, higher transcript accumulation for PFK was observed in healthy trees of cv. Leccino compared to healthy trees of cv. Ogliarola similar to HXK and PK (Figure 2). To the contrary, when we compared transcript accumulation of the non-regulatory glycolytic enzyme enolase in healthy trees between the two cultivars, we did not observe any power for discriminating both cultivars (Supplementary Figure S2). However, upon *Xf*-infection enolase transcript levels were decreased in both cultivars, while PFK transcript levels showed higher accumulation (Supplementary Figures S1 and S2). By calculating the sum of accumulated transcripts of the regulatory enzymes HXK, PFK and PK as a technical marker, named here *AdaptGlyc*, we observed that tree B cv. Leccino with highest presence of *Xf* could be discriminated from trees A and C with lower *Xf*-infection (Supplementary Figure S3). Tree C of cv. Ogliarola, which showed highest *Xf*-infection among all infected trees from both cultivars, could also be distinguished by using *AdaptGlyc* or by considering only transcript levels of PK (Supplementary Figure S3). Additionally, we searched for enzymes related to gluconeogenesis, such as PPi-dependent phosphofructokinase (PFP), phosphoenolpyruvate carboxykinase (PEPCK) and pyruvate orthophosphate dikinase (PPDK). However, we did not identify cultivar-specific transcript accumulation profiles in *Xf*-infected adult trees or a relation to the higher *Xf*-infection status of individual trees. 

The most striking difference between *Xf*-infected trees from both cultivars was obtained for transcript levels of enzymes involved in the ethanol fermentation pathway (PDC and ADH, Figure 2; Table S3). This was especially true for PDC, suggesting high differences in the release of acetaldehyde under *Xf*-infection. In cv. Leccino, healthy adult trees demonstrated already about two times higher transcript levels for PDC (1646.4 RPKM) and ADH (108.36 RPKM) compared to healthy trees of cv. Ogliarola (PDC: 737.8 RPKM; ADH: 51.89 RPKM). Furthermore, all *Xf*-infected trees of cv. Leccino showed high increase in PDC and ADH transcript levels that ranged for PDC from 3345.2 to 3948.7 RPKM and for ADH from 259.55 to 349.30 RPKM. On the contrary, *Xf*-infected trees of cv. Ogliarola displayed lower and dispersed transcript levels for PDC ranging from 499.7 to 1677.7 RPKM and for ADH from 77.37 to 130.49 RPKM. High transcript levels for PDC and ADH in *Xf*-infected trees of cv. Leccino linked to the higher transcript accumulation observed for HXK and PK under *Xf*-infection. However, in the tolerant cultivar transcript levels for PDC and ADH were not altered to the varying presence of *Xf*-cells. I.e., tree B of cv. Leccino showing highest *Xf*-infection did not demonstrate higher transcript levels for PDC and/or ADH than tree A or C. Nevertheless, in tree C of the *Xf*-susceptible cultivar Ogliarola, which showed highest *Xf*-infection, the higher transcript accumulation of HXK and PK in relation to healthy trees, also related to higher transcript levels for PDC (*Xf*: 1677.7 RPKM; healthy: 737.8 RPKM) and for ADH (*Xf*: 130.49 RPKM; healthy: 51.89 RPKM) though these were at much lower level than seen in cv. Leccino.

GEK1, a gene that was reported to be involved in ethanol/acetaldehyde tolerance [60,61,62], showed only low transcript levels and did not indicate cultivar-specific or *Xf*-infection status-related transcription. However, in infected trees of both cultivars we observed down-regulation of transcript levels for mitochondrial aldehyde dehydrogenase (mtALDH), an enzyme involved in acetaldehyde detoxification (Supplementary Figure S4). the gene representative for lactic fermentation, LDH, was expressed at low transcript level and did not show increase under *Xf*-infection in any of the trees from both cultivars (Figure 2; Table S3). 

### 3.3. Transcript Accumulation of β-cas and the Relation of Transcript Levels from COX and AOX Marked Xf-Infection in Trees of cv. Leccino, but Not in cv. Ogliarola

Despite the indicated increase of glycolysis in *Xf*-infected trees from cv. Leccino, we observed in these trees lower transcript accumulation for COX genes under the bacterial infection [healthy: 256.0 RPKM, *Xf*-infected: 225.11 RPKM, Table S3]. In contrast, AOX, as key gene of the cyanide-independent alternative respiration path, showed increased transcript levels upon *Xf*-infection [healthy: 46.2 RPKM, *Xf*-Infected: 76.91 RPKM]. Furthermore, we observed a peak for AOX in tree B, cv. Leccino, with highest *Xf*-infection [102.34 RPKM]. In cv. Ogliarola, mean values for transcript levels of both, COX and AOX, in healthy trees were similar to that observed in healthy trees of cv. Leccino. However, in the *Xf*-susceptible cultivar mean values of transcript levels for COX and AOX remained similar between healthy and *Xf*-infected trees. Nevertheless, tree C of cv. Ogliarola, which showed highest *Xf*-presence among all trees and also indicated increase in glycolysis (see text on top), a lower transcript level for COX (189.72 RPKM) was observed when compared to healthy trees (243.05 RPKM), that again as seen in cv. Leccino linked to higher transcript levels for PDC and ADH (see text on top). However, this decrease in COX transcript accumulation in tree C, cv. Ogliarola, was not accompanied by a higher transcript level for AOX (31.94 RPKM; mean value for cv. Ogliarola: 39.98 RPKM, Table S3).

**Figure 3:**
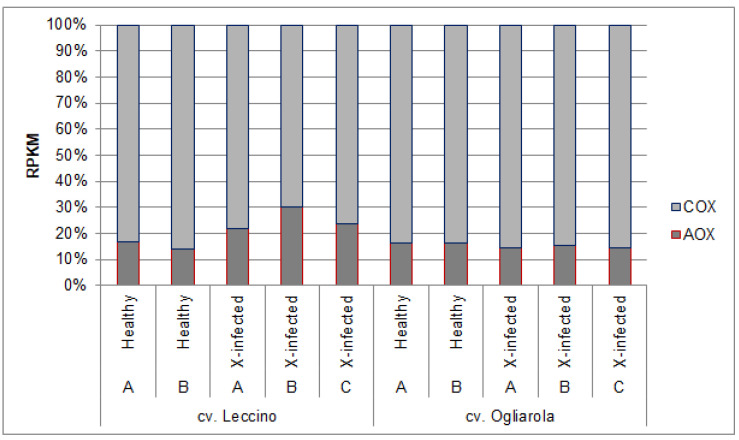
*Cytochrome c oxidase* (COX) and alternative oxidase (AOX) transcript accumulation (RPKM) in relation to each other (in %) from two healthy (A/B) and three *Xf*-infected (A/B/C) olive trees from cv. Leccino (*Xf*-tolerant) and cv. Ogliarola (*Xf*-susceptible).

In Figure 3**,** transcript accumulation for both respiratory enzymes, i.e., ‘total COX’ and ‘total AOX’, was set to 100% (RPKM values in Table S3). In this way, the distinct performance of the more tolerant cv. Leccino represented here by three selected individual trees could be marked: healthy trees from both cultivars and the *Xf*-infected susceptible cultivar showed almost identical relationship of transcript levels from COX and AOX (AOX: 14–17%), whereas the *Xf*-infected trees of cv. Leccino demonstrated relatively higher AOX transcript levels (22–30% AOX genes). We named this technical marker ‘rel-COX/AOX’.

β-Cyanoalanine synthase (β-CAS) is involved in cyanide detoxification. It can serve as an indicator for the presence of toxic cyanide levels, which typically were observed under abiotic and biotic stresses [63,64]. In healthy trees of both olive cultivars, transcript accumulation of this gene was at similar levels (cv. Leccino: 330.15 RPKM, cv. Ogliarola: 323.02 RPKM) (Table S3). However, upon *Xf*-infection, β-CAS showed highly increased transcript accumulation in cv. Leccino xylem tissue samples (484.72 RPKM) and the tree with highest *Xf*-infection (tree B, cv. Leccino) displayed the highest transcript level for β-CAS (543.97 RPKM) among all studied trees. In cv. Ogliarola, β-CAS showed no increase in tree A and only a slight increase in transcript levels for tree B, when *Xf*-infected trees were compared to healthy trees, and presented even the lowest level of transcripts in tree C despite this tree demonstrated highest *Xf*-infection among all infected trees from both cultivars. Of note, transcript levels of AOX and β-CAS showed high positive correlation across both cultivars (*R*^2^ = 0.82, significant at *p* < 0.05), indicative for their indirect relation via cyanide-mediated COX-inhibition. 

### 3.4. Transcript Levels of AOX and QDH Indicated a Link between Alternative Respiration and Quinic Acid Synthesis

The highest transcript level of AOX in the *Xf*-infected tree B in cv. Leccino, was connected to highest transcript accumulation for QDH. Accordingly, the low level of AOX transcripts in cv. Ogliarola in the highly *Xf*-infected tree C was linked to a low level of QDH transcripts. The relation of transcript levels from AOX and QDH across both cultivars was characterized by *R*^2^ = 0.60 (significant at *p* < 0.05), a correlation coefficient of medium strength (Table S3). QDH is involved in the synthesis of quinic acid, which was identified as promising marker from phenolic metabolism [36]. Quercetin-3-O-rhamnoside had been raised as another marker from phenolic metabolism that characterized a *Xf*-resistant Tunisian olive cultivar [36]. However, this was seen as a peculiarity for the Tunisian cultivar. In accordance, in all adult field-established trees from both Italian cultivars cv. Leccino and cv. Ogliarola, we found only very low transcript accumulation for 3-O-glycosyltransferase.

### 3.5. Xf-Infected Trees Indicated Gleichschaltung of AOX Genes’ Transcript Levels in Tolerant Cultivar, but Disturbed Transcript Profiles in Susceptible Cultivar

**Figure 4:**
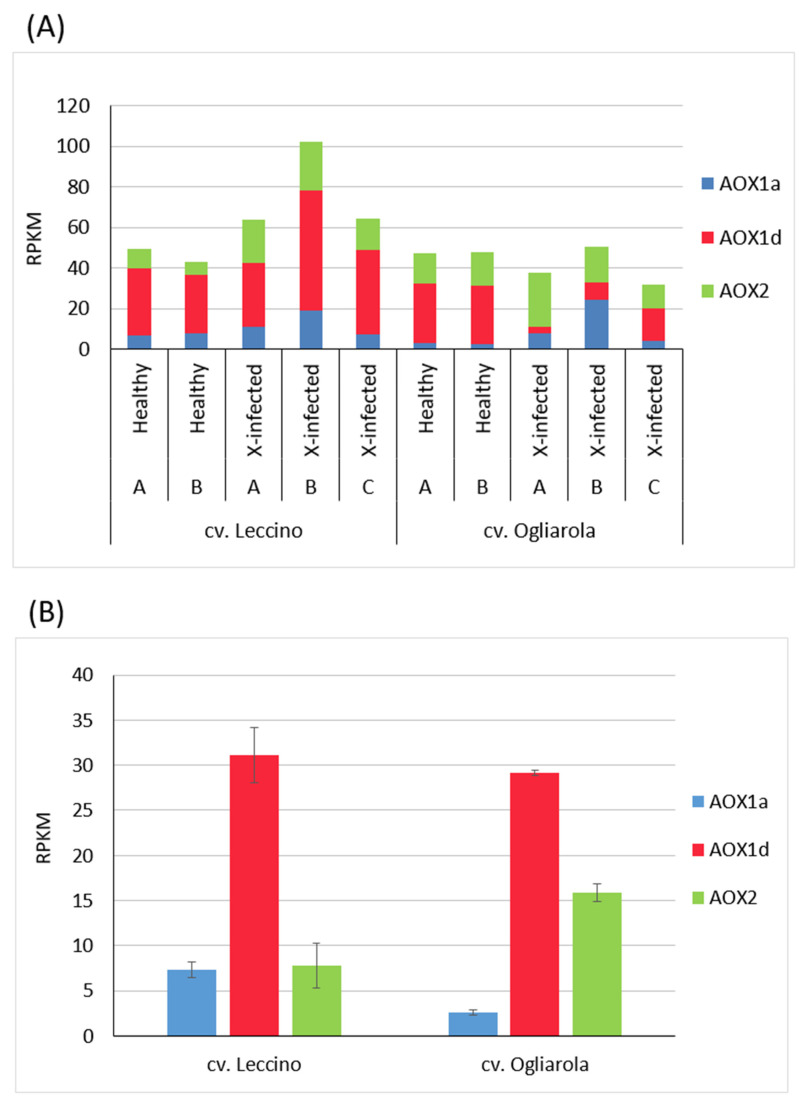
Gene expression of alternative oxidase (AOX) gene family members in olive trees. (**A**) Transcript accumulation of AOX1a, AOX1d and AOX2 from two healthy (A/B) and three *(Xf)*-infected (A/B/C) olive trees from cv. Leccino (*Xf*-tolerant) and cv. Ogliarola (*Xf*-susceptible). (**B**) Transcript levels of AOX1a, AOX1d and AOX2 in the mean of two healthy trees of cv. Leccino compared to two healthy trees of cv. Ogliarola. Bars represent standard errors.

In Figure 4A, transcript accumulation data presented in Figure 2 for AOX is separately shown for the transcribed individual AOX genes. Olive AOX is encoded by three AOX genes. Two belong to the AOX1 subfamily namely AOX1a and AOX1d, and one gene classified as AOX2 [65,66,67]. All AOX genes present in olive contributed to the transcript profiles identified for the five trees from cultivars Leccino and Ogliarola. Healthy trees of both cultivars with similar transcript levels of ‘total’ AOX were characterized by similarly dominating transcript accumulation of AOX1d among all AOX genes for both cultivars [31.09 RPKM in cv. Leccino, 29.15 RPKM in cv. Ogliarola (Figure 4B, Table S3)]. However, compared to cv. Leccino, cv. Ogliarola displayed lower AOX1a transcript levels (2.56 RPKM vs. 7.31 RPKM), but higher accumulation of AOX2 transcripts (15.84 vs. 7.79 RPKM) in the mean of both healthy trees (Figure 4B and Table S3). Upon *Xf*-infection, the cultivars revealed distinct differences in transcript accumulation patterns: in cv. Leccino all genes contributed to up-regulated total AOX transcript levels in an equilibrated way; i.e., none of the AOX genes was down-regulated beyond the level seen in healthy trees. In the tree with highest *Xf*-infection in cv. Leccino (tree B), all individual AOX genes, i.e., AOX1a, AOX1d and AOX2, showed higher levels of transcripts and together they displayed through this concerted up-regulation the highest level for total AOX (Figure 2) among the studied cv. Leccino trees. To the contrary, *Xf*-infection in cv. Ogliarola resulted for trees A and B in highly disturbed transcript accumulation profiles of individual AOX genes compared to healthy trees and to the profiles seen in cv. Leccino (Figure 4A). This change is mainly based on strong reduction in AOX1d transcript accumulation. At the same time, differential performance among individual *Xf*-infected trees was observed. In tree A of cv. Ogliarola an increase in AOX2 transcript accumulation was noticed that linked to a decrease in total AOX transcription, while in tree B higher AOX1a transcript accumulation was observed at unchanged total AOX transcript level. Of note, tree C from cv. Ogliarola, which showed the highest *Xf*-infection among all trees from both cultivars, displayed a similar pattern of AOX genes’ transcript accumulation as seen for healthy trees and also in *Xf*-infected trees of cv. Leccino, which was dominated by higher transcript accumulation of AOX1d. Nevertheless, the transcript level of AOX1d was down-regulated upon *Xf*-infection also in this tree (Figure 4).

### 3.6. Transcript Levels of α-, β-, and γ-Tubulin, E2F, SNF and TOR Indicated Down-Regulated Cell Cycle Activity as a Common Response to Xf-Infection and Signifcant Positive Correlation between AOX and Energy Consumption

**Figure 5:**
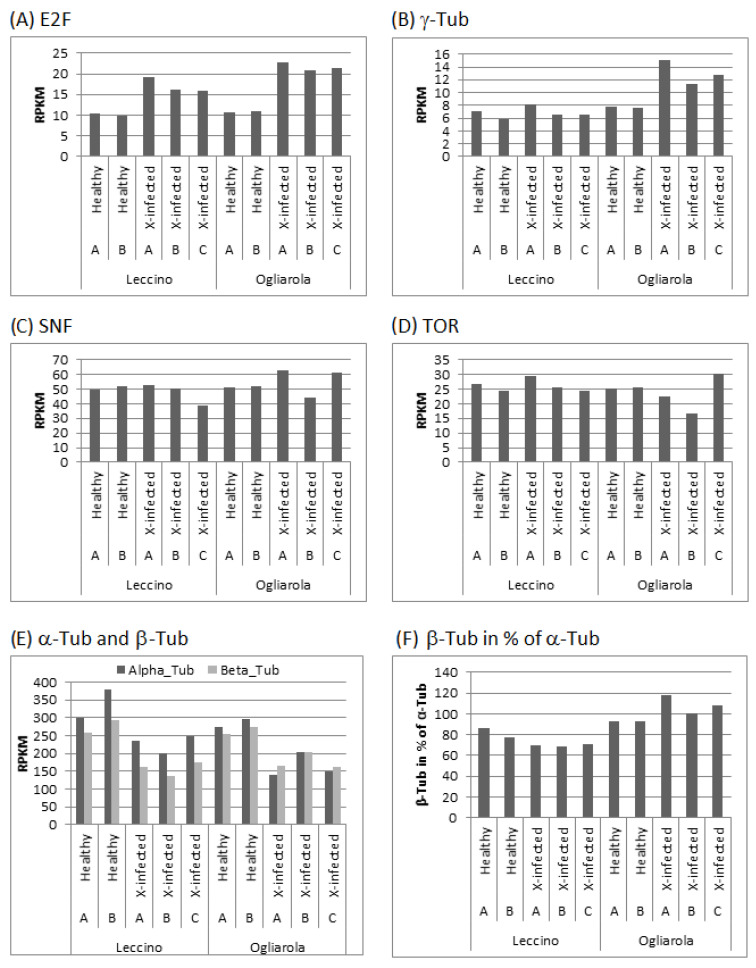
Transcript accumulation of genes involved in cell proliferation [E2F (**A**) and gamma-Tubulin (**B**)], energy consumption [SNF (**C**) and TOR (**D**)] and structural cell organization [alpha and beta-Tubulin (**E**,**F**)] in xylem tissues of mature stems from two healthy (A/B) and three *Xf*-infected (A/B/C) olive trees from cv. Leccino (tolerant) and cv. Ogliarola (susceptible); RPKM: Reads Per Kilobase per Million mapped reads, E2F: transcription factor E2F, SNF: Sucrose Non-Fermentable, TOR: Target Of Rapamycin.

Figure 5 demonstrates transcript accumulation of genes in the two healthy and three *Xf*–infected olive trees of cv. Leccino and cv. Ogliarola that relate to induction and initiation of cell proliferation, structural cell organization and energy consumption. In healthy trees, transcript accumulation of cell cycle-stimulating transcription factor genes E2F showed about the same level for both cultivars (Figure 5A, Table S3). In *Xf*-infected trees, this level is higher for both cultivars and seemed to be more increased for cv. Ogliarola. This observation is in conformity with observed higher transcript levels of γ-tubulin in cv. Ogliarola (Figure 5B, Table S3). Gamma-tubulin is known for its capacity to regulate centrosome dynamics and cell cycle progression and interacts with E2 promotor-binding factors [68]. This knowledge could be confirmed by our transcript analyses. We found significant high positive correlation between transcript levels of E2F and γ-tubulin (*R*^2^ = 0.93, *p* < 0.5) across all trees from both cultivars. However, transcript accumulation profiles of SNF (indicator for changes in energy consumption; higher values are indicative for higher energy consumption), TOR (signals change in cell cycle regulation; is activated when there is excess of energy) and alpha- and beta-tubulin (associate to structural cell organization; an increase links to higher cell cycle activity) in both cultivars clearly indicate that cell proliferation is in fact not promoted (Figure 5C–F, Table S3). To the contrary, clear down-regulation of alpha- and beta-tubulin transcript levels pointed to down-regulated cell cycle activity in all *Xf*-infected trees from both cultivars (Figure 5E). For cv. Leccino, this down-regulation of alpha- and beta-tubulin transcript accumulations is more pronounced in tree B, which corresponds in this cultivar to the tree with highest *Xf*-infection. Accordingly, we observed in tree B of cv. Ogliarola, which showed lowest *Xf*-infection among the three infected trees, lowest decrease in transcript levels of alpha- and beta-tubulin genes. Thus, down-regulation of both genes seems to indicate higher requirement for structural cell reorganization at down-regulated cell cycle activity as a common response to *Xf*-infection. Additionally, we observed in cv. Leccino higher transcript levels for total alpha-tubulin genes vs. transcript levels of total beta-tubulin genes in healthy and *Xf*-infected trees (Figure 5E and Figure 4F). The two healthy trees showed relative beta-tubulin transcript accumulation of 86% and 77%, while the three *Xf*-infected trees displayed more similar values from 68% to 71%. In contrast, in cv. Ogliarola *Xf*-infected trees indicated a less equilibrated change upon the bacterial stress from equal or even higher transcript accumulation for total beta-tubulin genes vs. total alpha-tubulin genes up to 118% (tree A). This changed relation between alpha- and beta-tubulin transcript levels indicated higher efforts in cv. Ogliarola to slow-down cell cycle activities [69]. Requirement for this higher effort to down-regulate cell cycling corresponds to the stronger efforts in this cultivar for cell cycle induction as signaled by transcript accumulation levels of genes for E2F (Figure 5A) and γ-tubulin (Figure 5B). Furthermore, the more stable transcript levels of SNF (Figure 5C) and TOR (Figure 5D) among the three *Xf*-infected trees of cv. Leccino compared to cv. Ogiarola indicate that the studied trees of the more tolerant cultivar could be characterized by intrinsically better equilibrated energy consumption in the presence of the bacterial stress despite the more intensive metabolic re-organization in the xylem tissue in primary and secondary metabolism as deduced from Figure 2. 

In this context, it might be of interest that AOX demonstrated across all trees from both cultivars significant positive correlation of medium strength to SNF (*R*^2^ = 0.64), while such a significant relation between AOX and energy consumption was not observed for one of the AOX genes alone. On the other hand, AOX1a, AOX1d and AOX2 are isozymes catalyzing the same reaction. Thus, differential AOX gene regulation between individual trees and/or cultivars might connect to a different physiological status. For example, we observed significant highly positive correlation between transcript levels of AOX genes and γ-tubulin only for AOX1d (*R*^2^ = 0.87), while transcript levels of AOX and all individual AOX genes revealed significant low to high correlation to transcript levels of NO-related genes (NR and/or ADH2/GSNOR). 

### 3.7. Under Xf-Infection, cv. Ogliarola Revealed Higher Transcript Levels for Fungal ITS2, RPB1 and Beta-Tub; Overall Bacterial 16S rRNA Transcript Levels Linked to Higher Xf-16S rRNA Transcription and Distinguished Individual Trees from Both Cultivars at Similar Xf-16S rRNA Transcript Levels

**Figure 6:**
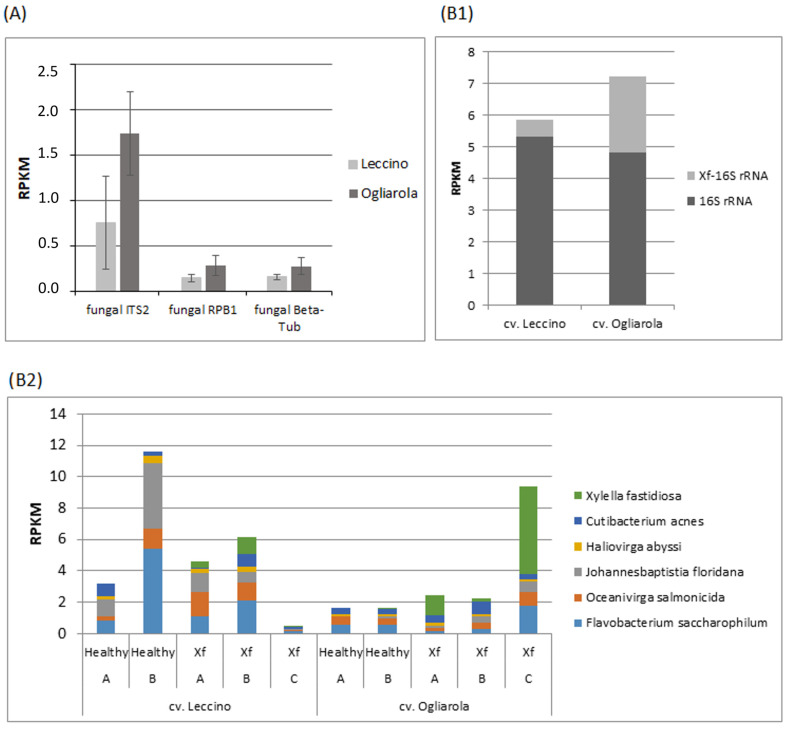
Transcript levels (RPKM) of fungal ITS2, RPB1 and Beta-Tub (**A**) and bacterial 16S rRNA (**B**) representing the fungal and bacterial communities in the mean of three *Xf*-infected olive trees from cv. Leccino and cv. Ogliarola. (**B1**) shows that mean values and the transcript level for *Xf*-16S rRNA is especially highlighted. Mean values and SE can be found in Table S3. (**B2**) displays 16S rRNA transcript levels of the five most abundant bacteria common in both cultivars in *Xf*-infected and healthy trees, including *Xf*.

Plant health might be affected by genotype-dependent viable microbiota or microbiota in the viable but non-culturable state [59]. Microbiomes as part of plant holobionts respond on the abiotic and biotic environmental changes that the whole organism is facing and challenges plant breeding [44]. Substrate competition within the microbial community with a special role of carbon sources is increasingly recognized as critical for microbiome structuring [70]. However, Giampetruzzi et al., [7] highlighted that the mechanisms of resistance against *Xf* likely reside on factors that are independent of the microbiome structure. Furthermore, microbiomes in general were suggested to contribute to metabolic stress relieve by buffering high amounts of carbon skeletons through carbon-consuming proliferation or secondary metabolism [40]. This view is not in contrast to the fact that increasingly isolated single components of microbiome structures can be successfully used to strengthen (or weaken, when applied as herbicides [71,72,73,74] plant fitness under various environmental conditions. Consequently, we hypothesized that transcript levels indicating higher carbon-consuming activities for the harbored fungal and/or bacterial communities as a whole, could mark the tolerant cultivar under *Xf*-infection. The *Xf*-infected trees of cv. Leccino and cv. Ogliarola studied here were grown in the same region under the same management regime [48]. Thus, transcript levels of RNA sequences, which identify fungal and bacterial microbiomes from these trees, can be assumed to reveal relevant differences in viable fungi and bacteria among the cultivars. Figure 6 represents transcript levels of fungal ITS2, RPB1 and Beta-Tub (Figure 6A) and bacterial 16S rRNA (Figure 6B) representative for fungal and bacterial communities in the mean of three *Xf*-infected olive trees from cv. Leccino and cv. Ogliarola (Table S3). We rejected the use of fungal 18S rRNA as inappropriate through false positives due to overlapping sequences with plant host transcripts. Overall, we identified fungal microbiomes mainly from *Ascomycota* and *Basidomycota* and bacterial microbiomes were dominated by Bacteriodetes, *Fusobacteriota*, *Cyanobacteriota*, *Actinobacteria* and *Protobacteria*. In Figure 6A it can be seen that cv. Ogliarola demonstrated higher overall transcript levels for the fungal community compared to the tolerant cv. Leccino. All three fungal identification markers pointed to similarly increased transcript levels from 181% for Beta-Tub to 195% for RPB1 and 229% for ITS2. We noted that this higher transcript accumulation of fungal Beta-Tub in the susceptible cultivar was clearly superior to the higher transcript level of 112% seen for the olive tree Beta-Tub gene in cv. Ogliarola compared to cv. Leccino. Genotype-dependent higher fungal tubulin activity can indicate increased cell cycle activity and/or structural cell re-organization linked to higher carbon consumption. Thus, in contrast to our hypothesis, the present observation showed that the fungal community as a whole employed by the *Xf*-susceptible cultivar demonstrated higher transcript levels of relevant sequences for carbon-consuming reprogramming than the tolerant cultivar.

**Figure 7:**
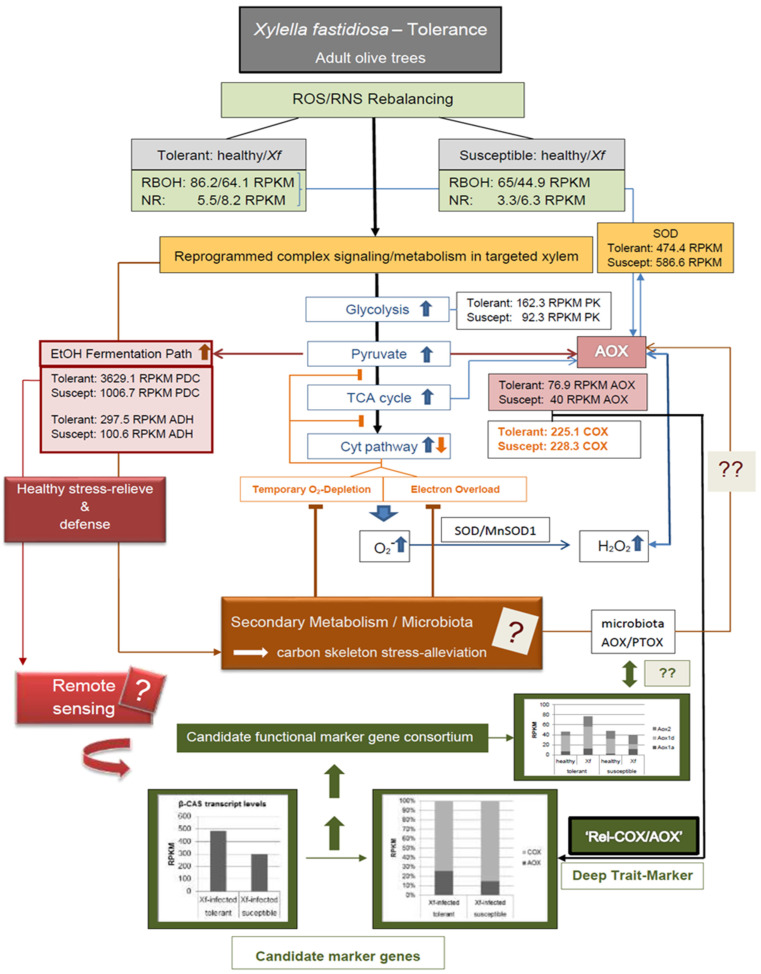
Validating utility of *CoV-MAC-TED* for identifying relevant deep marker-traits and candidate genes for functional marker development through transcriptome studies on *Xf*–affected adult olive trees with tolerant and susceptible performance—A proof-of-principles approach. Transcript levels in RPKM for SOD, PK, PDC, ADH, AOX and COX refer to mean transcript levels in infected trees (Table S3).

Figure 6(B1) seems to reveal also for the bacterial community in cv. Ogliarola higher transcript accumulation of 16S rRNA (123%). However, the higher overall transcript level of 16S rRNA of the whole bacterial community across the three *Xf*-infected trees did not significantly discriminate both cultivars and this was true with and without integration of the pathogen *Xf* (Table S3), indicating the importance of considering individual tree performance. Figure 6(B2) displays five most abundant and commonly identified bacteria from both cultivars for *Xf*-infected and healthy trees. It reveals that trees with the highest *Xf*-infection in each cultivar (tree B in cv. Leccino, tree C in cv. Ogliarola) displayed highest 16S rRNA transcript levels of the bacterial microbiota with and without the pathogen. Furthermore, tree B of cv. Leccino and tree A of cv. Ogliarola showed similar *Xf*-infection, but revealed clearly individual genotype-dependent transcript levels for the overall most abundant bacterial community. Thus, this observation strengthens the view that plant improvement by breeding and management strategies should consider directly or indirectly the plant holobiont nature [44,45,75]. Furthermore, these results support the hypothesis that bacterial pathogens might also contribute to carbon skeleton stress-relieve in favor to the host Figure 7 [40]. The higher 16S rRNA transcript levels for *Xf* observed in our study associated to the higher presence of the bacterial pathogen in the susceptible cultivar as it was demonstrated before by Giampetruzzi et al. [48]. However, it remains to be seen, whether higher transcript levels for the encountered bacterial 16S rRNA apart from the pathogen indicated the general higher amounts of bacterial cells, respectively more biomass. 

## 4. Conclusions and Perspectives

In Figure 7, we present a simplified scheme to summarize conclusions and applied perspectives based on the main results obtained by this study on healthy and naturally *Xf*-affected individual adult olive trees. 

The present study shows that transcriptome analyses of the *CoV-MAC-TED* gene set are useful also to characterize performance in terms of *Xf*-tolerance and *Xf*-susceptibility at the molecular level. ROS/RNS-rebalancing, indicated through down-regulated RBOH transcript levels and up-regulation of NO-producing NR transcripts, and down-regulated cell cycle activity were a common response to *Xf*-infection in both cultivars. However, RBOH/NR transcript level rebalancing in the tolerant cultivar indicated a clear link to glycolysis-driven strong up-regulation of the ethanol fermentation pathway and subsequent high PDC transcript accumulation. These observations suggest higher release of anti-microbial ethanol and acetaldehyde in the tolerant cultivar as a stress-alleviating response upon excessive carbon skeletons, which can be expected to interact with climate changes [76,77]. We propose that release of acetaldehyde might be explored for developing novel remote sensing technologies to improve real-time agro-management in view of the *Xf*-threat. 

At the same time, the scheme highlights the observed change in AOX transcript accumulation in *Xf*-infected olive trees in relation to more stable COX transcript levels and connects this to its complex and interacting regulation with redox and metabolic signaling and the multifunctional role of AOX associated to metabolic re-organization. The relation between transcript levels of both respiratory enzymes is a deep phenotype-trait, which can be technically marked for standardization through a number given in %, which can help to compare different systems and genotypes. The three individual trees of the tolerant olive cultivar could be discriminated from healthy and *Xf*-infected trees from both cultivars. One tree from cv. Leccino was characterized by highest total AOX with underlying concerted up-regulation of all three AOX genes and this was linked to highest *Xf*-infection among the three trees. These observations together encouraged our formerly developed view [13] that AOX sequence diversity control could serve as novel marker for on-field selection and early predicting *Xf*-tolerance. Moreover, because many studies from diverse research groups confirmed a critical role of AOX for tolerance performance against all types of abiotic and biotic stress conditions [8,10,11,40,78], these results also strengthen the perspective that more efficient and common regulation of the consortium of AOX genes can serve as trait for developing molecular marker for multiple-resilience. 

Furthermore, this scheme points to glycolysis-driven secondary metabolite synthesis and presence of microbiota as additional opportunities for rapid carbon-stress alleviation and the indirect role of AOX in this complex scenario. Our results indicated that the fungal community as a whole showed higher transcript levels for ITS2, RPB1 and Beta-Tub in *Xf*-infected trees of the susceptible cultivar than seen in the tolerant cultivar. This observation support the idea that AOX-related metabolic system equilibration in *Xf*-tolerant genotypes might reduce dependency from endophytes for fitness. A hypothesis that we think is valuable to be validated. Also, microbiota and parasites were shown to profit from own AOX or PTOX genes [79,80,81]. Thus, we suggest exploring furthermore the role of alternative respiration within plant holobiont system-components for plant stress-relief [40,82] and functional marker development. This might also be relevant for better understanding interaction between microbiota presence/absence and differential secondary metabolite synthesis [83,84,85]. However, it is of interest to note that Atkin et al. [86] observed AOX protein increase in relation to COX under low temperature. This did not happen, when plants had been treated with arbuscular mycorrhizal fungi (AMF). In this context, we should dare question whether breeding in favor of plant-AMF symbiosis makes sense or, to the contrary, would mean selection would favor weaker plant genotypes in terms of multiple-resilience *per se*, since dependency could become higher on bio-stimulants application that include mycorrhizal fungi. In Bharadwaj et al. [40], we proposed selecting seeds produced in parallel under organic and conventional farming by employing AOX-inhibition and presence of higher sucrose to obtain plants that are resilient under varying environmental conditions. Additionally, we demonstrated that it was possible to rank pea seeds early at germination by help of AOX-inhibition that associated to root rust disease tolerance. The involvement of AOX in pea germination and its link to higher temperature tolerance had subsequently been confirmed by help of calorespirometry [87,88].

In a theoretical approach, we had highlighted the importance to study the role of functional markers in target cells for man-defined agronomic traits that determines whole organism performance [41,42,44,45] and, consequently, developed a protocol for studying AOX genes at single cell level [89]. Furthermore, we studied isolated carrot AOX polymorphic genes on their effects on transformed yeast cell growth (Schizosaccharomyces pombe was used for transformation, which did not contain AOX genes [90] under mostly aerobic (cultivated with glycerol) or more anaerobic conditions (cultivated at higher glucose). By using calorespirometry to measure continuous heat rates, we observed differential effects of AOX1 and AOX2a genes early during growth initiation under aerobic conditions at mitochondrial respiration. However, identical effects of both genes on heat rates had been observed under anaerobic conditions characteristic for aerobic fermentation [91]. This indicated Gleichschaltung of AOX gene functionality related to growth behavior under more anaerobic conditions.

The present analyses indicated *Xf*-related down-regulation of cell cycle activities typical for cell reprogramming [11] and inducible by adding sucrose [40].This observation together with high aerobic fermentation, which is also known to occur in tree cambium cells dependent on the season [29] point to the involvement of cambial cells in the observed transcriptome changes upon *Xf*-infections. Therefore, to further strengthen deep trait-discovery we propose studying AOX gene family transcription in vascular cells with a special focus on cambial cells that should also involve tracing phenolic metabolites [92] and identification of fungal and bacterial endophytes [93]. This would enable cell-targeted isolation of coding and long noncoding RNAs of the consortium of AOX genes and subsequently allow applying complex substrate and regulator AOX affinity studies [94]. In this way, highly efficient functional marker development and further developed strategies for gene editing could be supported that can expect to improve *Xf*-tolerance and multiple-resilience. Nevertheless, strategies that aim to target AOX genes for genetic engineering, gene editing and therapeutic inhibition should carefully consider specific knowledge that was discussed in Aziz et al. [12] and Szibor et al. [80].

Remote sensing and real-time adapted agro-management strategies can be adopted to optimize plant fitness and secure plant survival [95,96]. Nevertheless, plant treatment strategies against *Xf*-infection would typically involve higher input of chemicals or bio-stimulants, are laborious and go along with higher costs [1,97]. Additional efforts to combat the *Xf*-threat are focusing on insect vectors [98,99] and should consider also higher ecological complexity [6,100]. However, the highest long-term impact and sustainability can be expected by multiple-resilient plant genotypes that do not require higher conventional or novel inputs to demonstrate optimized yield stability even in the presence of *Xf*. Therefore, priming [101] and breeding on *Xf*-tolerance for susceptible species of socio-economic importance were raised as valuable urgent requirements. Breeding was already shown a promising strategy, since differential genotype-dependent performance against this bacterial threat had been observed within susceptible species, such as *Vitis vinifera*, *Olea europaea* and diverse *Prunus* species [2,3]. Nevertheless, it was argued that tolerant genotypes will harbor *Xf* that might affect susceptible genotypes within the same region. Also, Quetglas et al. [1] highlighted the importance of maintaining diversity of planted cultivars to counteract the danger of monocultures developed for specific environmental threats, such as *Xf*. This view gets support from climate modeling in *Vitis arizonica*, which indicates climate dependency of *Xf*-resistance on temperatures and humidity [5]. Thus, it seems reasonable that sustainable plant production should consider substituting in the longer term susceptible genotypes by tolerant germplasm that demonstrates multiple resilience performance. At the same time, treatment with AMF-containing bio-stimulants could be explored to improve plant fitness of existing genotypes [40,82]. 

In Mohanapriya et al. [78] and Bharadwaj et al. [40], we proposed a low-cost and simple strategy to early identify genotypes with AOX-based higher resilience. This strategy considers the importance of efficient stress recovery indicated by rapidly reduced AOX expression after the stress-induced peak and measuring this dynamic by help of gradual AOX-inhibition. Thus, herein the AOX protein level is targeted. The strategy was successfully applied for germinating seeds. It was also shown that the priming effect could be measured in this way [78]. However, in case of established olive trees or other established plants, such as grapevine, where the usage of seeds is no option, similar test strategies could be developed by applying AOX-inhibition strategies on in vitro-systems under standardized stress-induced cell reprogramming conditions. Such strategies could be adopted to support field diagnosis and selecting resilient individual plants within cultivars and well-justified early decisions on *in-time* plant elimination.

Our concept of multiple-resilience [8,9,10,11,12,13,14,15] assumes that complex plant-environment interaction requires permanent acclimation and, additionally, that severe metabolic disturbances are more the rule than an exception. We hypothesized that lifelong adaptive robustness in resilient genotypes is provided by similar, early plant holobionts’ responses upon multiple stress that can be investigated at transcriptome level. Thus, our approach to link AOX transcript accumulation associated to Gleichschaltung of AOX family gene transcripts to multiple-resilience can be further validated by confirming repetitive relevance of these marker candidates along individual development and across genotypes and plant hologenomes from diverse species. Consequently, in Part II of this study, we explored transcriptomes from early hours to longer periods after experimental *Xf*-inoculation in tolerant and resistant Vitis plants from diverse trials and genetic origins (Arnholdt-Schmitt et al., under submission).

## 5. Dedication

All authors agreed to dedicate this study to ‘Independency and Collaboration—Learning about Peace from Tolerant Plants’.

## Data Availability

The raw data (BioProject number PRJNA316374) used to obtain the results presented in this paper were publicity available in GenBank (NCBI) by Giampetruzzi et al. [48] from Consiglio Nationale delle Ricerche, Istituto per la Protezione Sostenibile, coordinator Pasquale Saldarelli.

## Supplementary Materials

The following supporting information can be downloaded at: https://www.mdpi.com/article/10.3390/pathogens13030227/s1, Table S1. Gene members selected to study early cellular reprogramming in *Olea europaea* under *X. fastidiosa* infection; Table S2. Bioproject details of RNA-Seq experiment used to obtain the gene expression in olive; Table S3: Supplementary Tables File for Figures; Supplementary Figure S1: Transcript accumulation of phosphofructokinase (PFK) in xylem tissues; Supplementary Figure S2: Transcript accumulation of enolase in xylem tissues; Supplementary Figure S3: Glycolytic pathway technical marker (*AdaptGlyc*); Supplementary Figure S4: Transcript accumulation of mitochondrial aldehyde dehydrogenase *(mtALDH)* in xylem tissues.
