# Supplementary material for: Transcriptome Analyses in Adult Olive Trees Indicate Acetaldehyde Release and Cyanide-Mediated Respiration Traits as Critical for Tolerance against Xylella fastidiosa and Suggest AOX Gene Family as Marker for Multiple-Resilience
Source: Pathogens. 2024 Mar 5;13(3):227. doi: 10.3390/pathogens13030227 (PMC10975381; doi:10.3390/pathogens13030227)

Supplementary Figure S1. Transcript accumulation of phosphofructokinase (PFK) in xylem tissues of mature shoots of two healthy (A/B) and three *Xylella fastidiosa (Xf)*-infected olive trees (A/B/C) from cv. Leccino (*Xf*-tolerant) and cv. Ogliarola (*Xf*-susceptible).

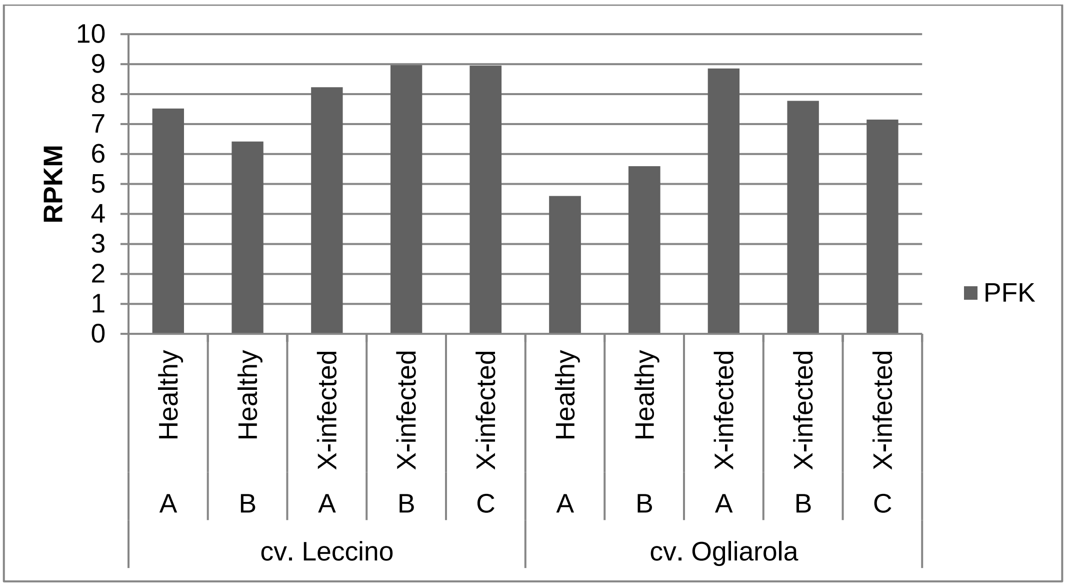

Supplement: Supplementary file 1 [file pathogens-13-00227-s001.zip › Supplementary Figure S1.pdf]
