# Supplementary material for: Transcriptome Analyses in Adult Olive Trees Indicate Acetaldehyde Release and Cyanide-Mediated Respiration Traits as Critical for Tolerance against Xylella fastidiosa and Suggest AOX Gene Family as Marker for Multiple-Resilience
Source: Pathogens. 2024 Mar 5;13(3):227. doi: 10.3390/pathogens13030227 (PMC10975381; doi:10.3390/pathogens13030227)

Supplementary Figure S3. Glycolytic pathway technical marker (*AdaptGlyc*) represented by the sum of accumulated transcripts of the regulatory enzymes HXK, PFK and PK.

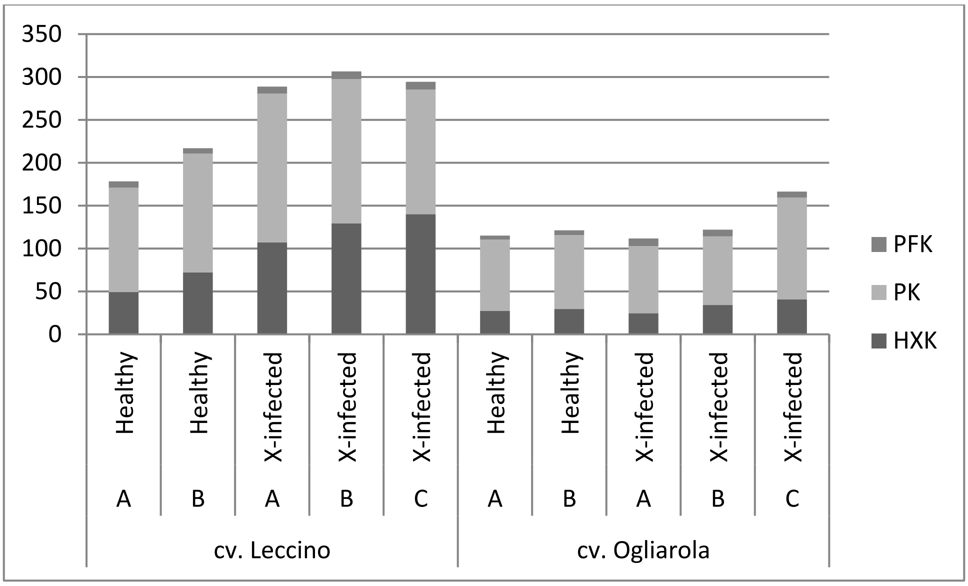

Supplement: Supplementary file 1 [file pathogens-13-00227-s001.zip › Supplementary Figure S3.pdf]
