# Supplementary material for: Transcriptome Analyses in Adult Olive Trees Indicate Acetaldehyde Release and Cyanide-Mediated Respiration Traits as Critical for Tolerance against Xylella fastidiosa and Suggest AOX Gene Family as Marker for Multiple-Resilience
Source: Pathogens. 2024 Mar 5;13(3):227. doi: 10.3390/pathogens13030227 (PMC10975381; doi:10.3390/pathogens13030227)
Supplement: Supplementary file 1 [file pathogens-13-00227-s001.zip › Supplementary Table S1.pdf]

Supplementary Table 1. Gene members selected to study early cellular reprogramming in *Olea europaea* under *X. fastidiosa* infection

Genes	*Olea europaea*	
	Gene member	Accession GenBank
Hexokinase (HXK)	Oe_HK-1a	XM_023026727.1
	Oe_HK-1b	XM_023037529.1
	Oe_HK-1c	XM_023038291.1
	Oe_HK-2a	XM_022998946.1
	Oe_HK-2b	XM_022987245.1
Phosphofructokinase (PFK)	Oe_PFK-1	XM_023041794.1
	Oe_PFK-2	XM_022990022.1
	Oe_PFK-3	XM_023016295.1
	Oe_PFK-4	XM_023034493.1
	Oe_PFK-5a	XM_023028776.1
	Oe_PFK-5b	XM_023015551.1
Enolase (Eno)	Oe_enolase_2	XM_022990965.1
	Oe_enolase_3	XM_023035000.1
Pyruvate kinase (PK)	Oe_PyrK-1	XM_023032857.1
	Oe_PyrK-2	XM_022990976.1
	Oe_PyrK-3	XM_023001818.1
	Oe_PyrK-4	XM_023006643.1
	Oe_PyrK-5	XM_023026232.1
	Oe_PyrK-6	XM_023035034.1
	Oe_PyrK-7	XM_023017181.1
Pyruvate Decarboxylase (PDC)	Oe_PDC1a	XM_023017909.1
	Oe_PDC1b	XM_023031688.1
	Oe_PDC2	XM_023029538.1
	Oe_PDC3	XM_022992236.1
Alcohol Dehydrogenase (ADH)	Oe_ADH1a	XM_023030093.1
	Oe_ADH1b	XM_022994954.1
	Oe_ADH2	XM_023017952.1
Lactate Dehydrogenase (LDH)	Oe_LacDH_A	XM_023025336.1
	Oe_LacDH_B	XM_023033175.1
Cytochrome c oxidase (COX)	Oe_COX3-L	XR_002700606.1
	Oe_COX5b-1a	XM_023034449.1
	Oe_COX5b-1b	XM_022987766.1
	Oe_COX5b-1c	XM_023033942.1
	Oe_COX5b-1d	XM_022996582.1
	Oe_COX5C-1a	XM_022998118.1
	Oe_COX5C-1b	XM_022999354.1

	Oe_COX5C-1c	XM_023033650.1
	Oe_COX5C-1d	XM_022986343.1
	Oe_COX5C-4	XM_023039691.1
	Oe_COX6a-1	XM_023041429.1
	Oe_COX6a-2	XM_022985977.1
	Oe_COX6b-1a	XM_023020374.1
	Oe_COX6b-2a	XM_023007365.1
	Oe_COX6b-2b	XM_023032334.1
	Oe_COX6b-1b	XM_022989391.1
	Oe_COX6b-2c	XM_023016680.1
Sucrose non-fermenting protein kinase (SNF)	Oe_SNF1_1	XM_022994974.1
	Oe_SNF1_2	XM_023002181.1
	Oe_SNF1_3	XM_022989140.1
	Oe_SNF1_4	XM_023016316.1
Target Of Rapamycin protein kinase (TOR)	Oe_TOR1	XM_023041148.1
	Oe_TOR2	XM_022989596.1
Transcription factor E2F	Oe_E2FA1	XM_023010913.1
	Oe_E2FA2	XM_022986067.1
	Oe_E2FA3	XM_023002019.1
	Oe_E2FB1	XM_023028000.1
	Oe_E2FB2	XM_023043124.1
	Oe_E2FB3	XM_023013542.1
Cu/Zn - Superoxide Dismutase (Cu/Zn-SOD)	Oe_Cu/Zn-SOD1	XM_023008673.1
	Oe_Cu/Zn-SOD2	XM_022993491.1
	Oe_Cu/Zn-SOD3	XM_023002054.1
	Oe_Cu/Zn-SOD4	XM_022995793.1
Fe - Superoxide Dismutase (Fe-SOD)	Oe_Fe-SOD1	XM_023032589.1
	Oe_Fe-SOD2	XM_023032593.1
Mn - Superoxide Dismutase (Mn-SOD)	Oe_MnSOD1	XM_023011415.1
	Oe_MnSOD2	XM_023009720.1
NADPH oxidases (RBOH)	Oe_RBOH-A1	XM_022989693.1
	Oe_RBOH-A2	XM_023025524.1
	Oe_RBOH-C	XM_022986908.1
	Oe_RBOH-E	XM_023005430.1
	Oe_RBOH-H	XM_023037344.1
Alcohol dehydrogenase2 / S-nitrosoglutathione reductase (ADH2_GSNOR)	Oe_ADH2_GSNOR-1	XM_023034886.1
	Oe_ADH2_GSNOR-2	XM_023018821.1
Alternative oxidase (AOX)	Oe_Aox1a	XM_023000218.1
	Oe_Aox1d	XM_023025579.1
	Oe_Aox2	XM_023001633.1
β-Cyanoalanine synthase (β-CAS)	Oe_B-Cas1	XM_023015002.1
	Oe_B-Cas_A	XM_023016718.1

	Oe_B-Cas_B	XM_023003682.1
	Oe_B-Cas_C	XM_023019182.1
	Oe_B-Cas_D	XM_023000461.1
Nitrate reductase (NR)	Oe_NR-1	XM_023013960.1
	Oe_NR-2	XM_023007920.1
	Oe_NR-3	XM_023032166.1
D-aminoacyl-tRNA deacylase (GEK1)	Oe_GEK1	XM_023012777.1
Mitochondrial Aldehyde Dehydrogenase (mtALDH)	AlDH_2B4	XM_023034099.1
	AlDH_2B7	XM_022995883.1
Quitinate dehydrogenase (QDH)	Oe_QDH1	XM_022998714.1
	Oe_QDH2	XM_023006041.1
	Oe_QDH3	XM_022989008.1
Anthocyanidin 3-O-glucosyltransferase (3o-GT)	Oe_3o-GT	XM_022997285.1
Alpha tubulin (Alpha_Tub)	Oe_Alpha_Tub1	XM_023028584.1
	Oe_Alpha_Tub2	XM_022993342.1
	Oe_Alpha_Tub3	XM_023010521.1
	Oe_Alpha_Tub4	XM_023040402.1
	Oe_Alpha_Tub5	XM_023012263.1
	Oe_Alpha_Tub6	XM_023002416.1
	Oe_Alpha_Tub7	XM_022995839.1
Beta tubulin (Beta_TUB)	Oe_beta_TubA	XM_023024284.1
	Oe_beta_TubB	XM_023028953.1
	Oe_beta_TubC	XM_022988543.1
	Oe_beta_TubD	XM_023016355.1
	Oe_beta_TubE	XM_023007159.1
	Oe_beta_TubF	XM_022989745.1
	Oe_beta_TubG	XM_023011802.1
	Oe_beta_TubH	XM_022998136.1
	Oe_beta_TubI	XM_023027461.1
	Oe_beta_TubJ	XM_023018663.1
	Oe_beta_TubK	XM_023023609.1
	Oe_beta_TubL	XM_023043247.1
	Oe_beta_TubM	XM_023037140.1
	Oe_beta_TubN	XM_022989736.1
	Oe_beta_TubO	XM_023036069.1
Gamma tubulin (Gamma_Tub)	Oe_Gamma_Tub	XM_023026882.1

Pyrophosphate--fructose 6-phosphate 1-phosphotransferase (PFP)	Oe_PFP_alpha1	XM_023023240.1
	Oe_PFP_alpha2	XM_023011969.1
	Oe_PFP_alpha3	XM_022999007.1
	Oe_PFP_alpha4	XM_023018258.1
	Oe_PFP_Beta1	XM_022996590.1
	Oe_PFP_Beta2	XM_023014523.1
	Oe_PFP_Beta3	XM_023036508.1
Phosphoenolpyruvate carboxykinase (PEPCK)	Oe_PEPCK1	XM_023016928.1
	Oe_PEPCK2	XM_022993827.1
Pyruvate orthophosphate dikinase (PPDK)	Oe_PPDK	XM_022988170.1
