# Supplementary material for: Transcriptome Analyses in Adult Olive Trees Indicate Acetaldehyde Release and Cyanide-Mediated Respiration Traits as Critical for Tolerance against Xylella fastidiosa and Suggest AOX Gene Family as Marker for Multiple-Resilience
Source: Pathogens. 2024 Mar 5;13(3):227. doi: 10.3390/pathogens13030227 (PMC10975381; doi:10.3390/pathogens13030227)
Supplement: Supplementary file 1 [file pathogens-13-00227-s001.zip › Supplementary Table S2.pdf]

Supplementary Table 2. Bioproject details of RNA-Seq experiment used to obtain the gene expression in olive.

**Species**	**BioProject accession**	**Experiment details**	**Tissue/Cell type**	**Biological replicates**	**Reference**
Olea europaea	PRJNA316374	- Transcriptome profiling of healthy and *Xylella fastidiosa* (*Xf*)-infected olive trees from cv. Leccino (*Xf*-tolerant) and cv. Ogliarola salentina (*Xf*-susceptible).<br><br>- Samples were collected in field. Xf-infected 25 year-old trees of both cultivars originated from the same Xf-affected grove grown under the same management regime.<br>Xf-infected and healthy trees showed comparable age and phenological state.	Xylem of mature shoots	Two healthy olive trees (control condition) and three *Xf*-infected olive trees (infected condition) corresponding to a total of 5 trees for from each cultivar.	Giampetruzzi et al. [48]
